# Implementation of climate adaptation in the public health sector in Europe: qualitative thematic analysis

**DOI:** 10.1093/eurpub/ckad218

**Published:** 2023-12-14

**Authors:** Grace A Turner, Francesca de’Donato, Annechien D Hoeben, Zuzana Nordeng, Samantha Coleman, Ilona M Otto, Shakoor Hajat, Sari Kovats

**Affiliations:** Department of Public Health, Environments and Society, London School of Hygiene and Tropical Medicine, London, UK; NIHR Health Protection Research Unit in Environmental Change and Health, London School of Hygiene and Tropical Medicine, London, UK; Department of Epidemiology, Lazio Regional Health Service - ASL Roma 1, Rome, Italy; Wegener Center for Climate and Global Change and Institute for Environmental Systems Sciences, University of Graz, Graz, Austria; Department of Research Administrative Support, Norwegian Public Health Institute, Oslo, Norway; Department of Public Health, Environments and Society, London School of Hygiene and Tropical Medicine, London, UK; Wegener Center for Climate and Global Change and Institute for Environmental Systems Sciences, University of Graz, Graz, Austria; Department of Public Health, Environments and Society, London School of Hygiene and Tropical Medicine, London, UK; NIHR Health Protection Research Unit in Environmental Change and Health, London School of Hygiene and Tropical Medicine, London, UK; Department of Public Health, Environments and Society, London School of Hygiene and Tropical Medicine, London, UK; NIHR Health Protection Research Unit in Environmental Change and Health, London School of Hygiene and Tropical Medicine, London, UK

## Abstract

**Background:**

Adaptation, to reduce the health impacts of climate change, is driven by political action, public support and events (extreme weather). National adaptation policies or strategies are limited in addressing human health risks and implementation of adaptation in the public health community is not well understood.

**Aim:**

To identify key issues in climate change adaptation implementation for public health in Europe.

**Methods:**

Key informant interviews with decision-makers in international, national and local city governments in 19 European countries. Participants were recruited if a senior decision-maker working in public health, environmental health or climate adaptation.

**Interviews addressed:**

Barriers and levers for adaptation, policy alignment, networks and evidence needs.

**Results:**

Thirty-two interviews were completed between June and October 2021 with 4 international, 5 national and 23 city/local government stakeholders. Respondents reported inadequate resources (funding, training and personnel) for health-adaptation implementation and the marginal role of health in adaptation policy. A clear mandate to act was key for implementation and resource allocation. Limited cross-departmental collaboration and poor understanding of the role of public health in climate policy were barriers to implementation.

**Conclusions:**

Across Europe, progress is varied in implementation of climate adaptation in public health planning. Providing appropriate resources, training, knowledge mobilization and supporting cross-departmental collaboration and multi-level governance will facilitate adaptation to protect human health.

## Introduction

The latest Intergovernmental Panel on Climate Change (IPCC) reports confirm that climate change is already affecting human health.^1^ In Europe, exposure to heatwaves has increased by nearly 60% from 2000 to 2009 compared with 2010–19.^2^ A range of climate adaptation policies to address climate risks to health are being implemented within the European Union (EU) at both national and local government levels. Policy development has been driven by high level political action (EU Adaptation Strategy),^3^ national climate legislation, public support and climate activism. Following the Seventh Ministerial Conference on Environment and Health in Budapest in 2023, World Health Organization (WHO-EURO) Member States have committed to accelerating a just transition towards resilient, healthy, equitable and sustainable societies and prioritize action on health challenges related to climate change.^4^ As part of the previous Ostrava Ministerial declaration, Member States committed to developing National Portfolios of Actions on Environment and Health (NPs) with most member states using NPs to report existing environment and health policies and as a tool for ‘health and environment in all policies’.^5^ The European Climate Law 2021 commits EU Member States to address adaptation.^6^ The EU Adaptation Strategy published in 2021, addresses health to some extent and established the European Climate and Health Observatory (ECHO) Partnership.^3^ WHO supports countries to implement resilient and low-carbon sustainable health systems through the ATACH framework^7^ and the WHO working group on Health in Climate Change supports action for health in the WHO European Region.^8^

Overall, progress in adaptation is slow and the health sector is behind other sectors in terms of adaptation planning.^9^ Following Covid-19, the NextGenerationEU Recovery and Resilience Fund was launched for supporting the economic recovery of EU Member States focusing on a green transition and resilient health care systems and response.^10^ However, only 6% of these commitments are dedicated to climate adaptation and even less include climate actions, which benefit health.^11^ Nearly all EU countries have national adaptation policies but few include health-adaptation priorities or goals. The extent to which adaptation policies are implemented in public health and the health sector is unclear.^12^

Local governments play an increasingly important role in implementing adaptation for health. The Carbon Disclosure Project (CDP) and International Council for Local Environmental Initiatives (ICLEI) found that in 2021, 118/150 European cities reported climate change as a threat to public health or health services in city-level assessments.^2^ The EU Adaptation Strategy acknowledges that local authorities are best equipped to implement climate adaptation action for health due to their knowledge of population characteristics, local climate hazards and community engagement.^3^ However, most adaptation measures are still technologically focused and fail to consider health.^13^ Many barriers to implementing adaptation are the same barriers for public health action in general.

This study aims to identify key issues in climate adaptation implementation for public health in Europe and to explore the barriers and policy levers through an inductive thematic analysis of key decision-maker interviews.

## Methods

### Research design

To explore implementation of adaptation measures for public health in Europe, we chose a qualitative research design used to identify key issues through key informant semi-structured interviews with decision-makers. This study used a phenomenological approach through inductive thematic analysis to identify patterns and themes deriving directly from the data without pre-existing expectation of participant perceptions of barriers or facilitators for implementing climate and health adaptation.

### Study population

Key informant interviews were undertaken with decision-makers working in international, national or local governments. Participants were recruited using a purposive maximum variation sampling approach from organizational networks. Additional participants were identified through snowballing. In total, 39 potential participants were emailed to be involved in this study. Interviewees were required to be decision-makers in either European Commission, national or city-level government and be in a senior or leadership role in one of the following departments, agencies or institutions: Environmental Health, Public Health or Adaptation. We interviewed decision-makers to broaden the scope for discussion on implementing climate and health-adaptation actions that may not be specific to more formalized policies. We aimed to have a balanced distribution across the EU region and also included Norway and the UK.

### Data collection

Individual semi-structured interviews were conducted and recorded through videoconferencing between June and October 2021. Interviews were 45 min, conducted in English or the local language and transcribed into English. Interviews were carried out by the following researchers—G.A.T. (MSc), A.D.H. (MSc), F.d.D. (PhD) and S.K. (PhD). Interviewers have a research background in climate change and health. Each interview used a topic guide (Supplementary material S1); participants were asked about their knowledge and interest in climate-health adaptation, capacity to implement action, evidence needs, networks and collaborative working and the broader public health agenda. All transcripts were checked by the original interviewer and on reviewing transcript content to familiarize with the data, it was determined that data saturation had been reached.

### Data analysis

Thematic analysis with open coding was performed with codes being generated inductively. Themes and sub-theme codes were identified and refined throughout analysis (full list in Supplementary material S2). To ensure the quality of analysis, researchers coded 25% of transcripts separately and compared outputs. Following on from this, G.A.T. coded the remaining transcripts. All transcripts were analysed in NVivo R1.7 software and the framework matrix of summarized data, quotations and codes by theme were stored in Microsoft Excel. This research follows the Consolidated criteria for Reporting Qualitative Research Checklist (checklist in Supplementary material S3).

### Ethics approval

The London School of Hygiene and Tropical Medicine Research Ethics Committee approved this study in June 2021 (Ref No. 25707) including researcher’s appropriate training and experience in qualitative methods. Participants were informed of the study aims and objectives and gave written consent for interviews to be recorded and transcribed (Supplementary material S4).

## Results

Key informant interviews were undertaken with decision-makers across 17 EU countries (Cyprus, Spain, Ireland, Finland, Lithuania, Belgium, the Netherlands, Sweden, Latvia, Italy, Estonia, Austria, Croatia, France, Germany, Hungary and Denmark) and Norway and the UK (EU+). Thirty-two interviews were completed (table 1) including 12 participants who had a senior role in Environmental Health, 11 Adaptation Leads and 9 Directors of Public Health. Twenty-three participants were city-level decision-makers, five were from national government and four interviewees were from the European Commission.

**Table 1:** Number of participants in interviews by role

Government role	City	National	International	Total
Environmental health (senior minister or lead)	6	3	3	12
Climate adaptation lead	9	1	1	11
Director of Public Health	8	1	–	9
Totals	23	5	4	32

Key themes on adaptation and public health (table 2) are discussed in more detail below.

**Table 2:** Key interview themes and sub-themes

Theme	Sub-theme
Aligning the climate and health agenda	Health in all policies
Power and governance structure	City-level policy remit for health adaptation
	Governance constraints
	Public acceptance/support
Funding needs and capacity requirements	Financial implications
	Resources and Implementation capacity
Inter-sectoral working and cross-collaboration	Cross-departmental working
	Departmental silos
Operational knowledge and data mobilization	Knowledge limitations
	Implementation strategy and operational policy

### Aligning the climate and health agendas

#### Health in all policies

Nearly all interviewees stated the importance of consulting and including health professionals in implementing climate adaptation strategies and interventions for health. Many participants referenced health as important for prioritizing climate adaptation action in other sectors, as this quote indicates: ‘I do think the penny is dropping about the importance of health as a lever for climate action’ Adaptation lead—City level. Most participants supported that the ‘health in all policies’ approach and creating the ‘bigger picture’ for health within other departments is necessary for climate and health adaptation. Many respondents indicated health is rarely included in climate adaptation planning but wanted to address health issues in future planning, as mentioned by one respondent: ‘The main goal is to make sure that health is taken into account in climate adaptation policy. As we see that in most departments on climate adaptation, it’s not considering health at all’ Environmental health—City level.

### Governance for adaptation

#### City-level policy remit for health adaptation

Having a national or city-level mandate to act was considered a key lever for health-adaptation implementation and resource allocation. Some city government representatives highlighted cities to be leading on climate action, which went beyond national policy or position, e.g. declaring climate emergencies, although these activities were not specifically addressing health. Often many recalled having limited power to advocate or focus specifically on adaptation for health, with national governments viewed as typically better resourced to address health adaptation. Most city-level participants interviewed called for a local statutory, specific remit on delivering climate and health action with clear leadership from national government, adequate funding and capacity allocation to close the gap between national and local implementation. ‘So there is a need for national and international focus, especially when it comes to these areas that are still quite new to us at the local level, on how to deal with it. There is a need for a national and international lead and focus’ Adaptation lead—City level. Limited engagement between levels of government levels was reflected by an international decision-maker ‘I think that our outreach, for example, local authorities is a bit less intense. We don’t have this sort of direct interactions with subnational levels of governance’ Environmental Health—International level. Horizontal engagement was more apparent, as many city representatives highlighted good, well-established partnerships between cities.

#### Governance constraints

A key implementation barrier acknowledged by city-level interviewees was following the political direction of European and national government, compounded by changing political priorities following electoral cycles. ‘We have the capacity to implement it, but logically we always agree with the political leaders…there is a political direction that is established by the general lines of action’ Public health—City level. To address this, local government participants suggested engaging political leaders on the importance of responding to the health impacts from climate change and demonstrating the multiple benefits of implementing climate adaptation action, e.g. public health, economic and biodiversity/environmental benefits. Participants identified key opportunities for action followed regional climate events (e.g. heatwaves), which disrupted services and impacted health. ‘I was working with policymakers from local down to the national level, and they would only pay attention and really implement [adaptation] measures when they see evidence’ Environmental health—International level. Political leadership can support or undermine climate-health adaptation reflecting the difference experiences of respondents and dependency on high level political support in their country.

#### Public acceptance and support

Public support was reported as crucial for implementing climate adaptation as it helped raise importance in government as indicated here: ‘quite clearly the political pressure is also there. People are incredibly worried about climate change, and I think politicians have picked it up’Adaptation lead—International level. However, although public support for mitigation (emissions reduction) can be high, respondents highlighted that there is limited public awareness of the health impacts from climate change and therefore less understanding of the need for adaptation to benefit health. ‘We will need additional investment in terms of people, and we need an ongoing investment in terms of research. But the greatest capacity gap is really in public awareness and political commitment’ Environmental Health—City level. Some interviewees stressed the need to engage with the public early and demonstrate local benefits to minimize government distrust to act in the public interest. As one example shows: ‘there's a big distrust of Council, so it's working closely with people…And that was the motivation for me’ Adaptation lead—City level.

### Funding needs and capacity requirements

#### Financial implications

A barrier to implementing adaptation measures for health was insufficient resources and funding according to nearly all participants. This was particularly evident for local government. City-level health professionals say adaptation is not prioritized in the public health budget, and adaptation is viewed as an extra activity rather than integrated into core public health measures. However, others viewed accounting for health costs could encourage funding: ‘But because you don't see a direct benefit, it's hard to entice policy makers or funding into something which you don't really know what is beneficial. But if you think of health, that's the added value, because if it benefits your health, it's a benefit anyway’ Environmental health—International level.

#### Resources and implementation capacity

Respondents cited capacity limitations as a barrier to implementation, including a need for additional personnel. Specifically, participants called for improved training of public health professionals on the health impacts of climate change to facilitate collaboration with other departments. City-level participants raised concerns about the expectations for implementing health-adaptation interventions due to staff capacity already maximized to deliver core priorities. ‘There's often maybe one person or a maximum of two people working on heat or adaptation in general in a city government. So, it's an additional program that we're proposing on cities and they think it is great but also need capacity or someone who will help them do it’ Adaptation lead—City level. Some study participants from city-level governments reported having introduced roles for adaptation implementation, although these roles do not fall within the health departments.

### Importance of inter-sectoral working

#### Inter-sectoral working

The lack of interdisciplinary and cross-departmental working is a barrier to climate and health adaptation which appeared in almost all responses: ‘Especially in terms of environmental issues, of climate change related to health. They sort of run…parallel…No one's ever asked the other one what they were doing because I think in health there is much more related to climate or extreme events or environmental risks that is being done, which is never associated to the policy of climate change, which is a shame’ Environmental health—International level. Furthermore, participants commented on the role of funding calls to prioritize an interagency approach to align research and policy across the broader climate and health agenda, e.g.: ‘research in this area tends to get funded for one specific area and not that interagency approach. So I feel very strongly about that. Interagency is where the real action can happen’ Adaptation lead—City level*.* A few local interviewees gave examples of successful multi-sectoral working with research, industry and community stakeholders to develop strategies, e.g. Healthy City Action Plan.

#### Adaptation vs. mitigation

A reported barrier to effective health-adaptation implementation is the disconnect between climate mitigation and climate adaptation. The integration of these streams is necessary in some policy areas, but it is challenging: ‘what are the core benefits for health and the environment? Because it's all interactive and even within one Ministry, you have the silos, you have the mitigation people, you have the adaptation people, and we from the health sector’ Environmental health—National level. Prioritizing knowledge sharing and collaboration between those working in adaptation and those in public health is essential: ‘we can see that the mitigation, climate and health community is already well-established in terms of co-operation. But adaptation, it's not at that level, not because of a lack of identifying a research topic itself, I would say the contact between the two communities [adaptation and public health] language, it's still quite different. There is the co-operation but it's not yet at the same level’Environmental health—International level.

### Operational knowledge and data mobilization

Participants’ understanding and knowledge on climate and health adaptation varied based on governance level, role and region. Many respondents commonly referred to knowledge gaps, with the majority not comprehending climate risks to health and wellbeing. Although decision-makers had awareness of relevant evidence, it was typically not being operationalized into action, reasons being limited time, capacity and access. Respondents commonly used WHO reports as they had difficulty in extracting the key messages from academic research calling for a need to strengthen partnerships with academia and research: ‘I think there could be a stronger connection to academic work, but at the same time, implementable; how to use and do research on a local level’ Adaptation lead—City level. Participation in established public health networks, e.g. WHO Healthy City Network was reported as an opportunity for knowledge sharing between cities due to the interdisciplinary approach, access to data and evidence dissemination. ‘you see differences in how the cities within the Healthy City network implement that ambition…it's always about this more holistic and interdisciplinary and integrated way of working’ Public Health—City level.

## Discussion

### Main findings

We identified varied knowledge and progress in the implementation of adaptation to climate change in the public health sector across EU+ but widespread agreement for a health in all policies approach throughout government. Inadequate resources (funding, training and personnel) and limited attention to climate and health-adaptation policy were common challenges. International and national government leadership and support for city-level statutory, specific remits are viewed as critical to facilitate implementing climate adaptation for public health. Furthermore, political direction and electoral cycles can both undermine or support climate adaptation for health. Decision-makers aim to increase political engagement through demonstrating the benefits of climate adaptation for health, the economy and the environment. Public support and trust was viewed as important for climate action, however, public awareness of the health risks of climate change and the role of adaptation for health is not well understood. Almost all interviewees reported limited inter-sectoral collaboration between and across governance levels, as well as between mitigation, adaptation and public health departments as a barrier, suggesting funding opportunities to prioritize interagency approaches. Improving the training of public health professionals on the impacts from climate change on health was important for facilitating collaboration with other departments and implementing targeted adaptation action. Although, city-level participants are concerned about staff responsibilities and capacity for additional priorities beyond current core commitments without additional resource. Whilst many interviewees were aware of evidence, this was not routinely operationalized into action due to access, time and capacity constraints. This could be done through strengthening partnerships between government and academia and collaboration between local government through climate and health networks to facilitate implementing evidence-based climate and health-adaptation action more successfully.

### Existing literature

Previous qualitative studies exploring implementation barriers and enablers of climate adaptation and health align with this article’s findings.^14^^,^^15^ Inadequate funding is consistently reported as a significant barrier to the implementation of activities addressing the climate and health agenda. Globally, adaptation funding for health systems constitutes <1% of global funding for climate change adaptation.^16^ However, increased resources are unlikely to significantly improve the implementation of climate and health-adaptation policy without adequate staff training and education. As recommended by the European Environment Agency and endorsed by nearly 100 key European stakeholders; integrating climate resilience and climate-health literacy among health professionals as well as investing in interdisciplinary education is essential.^13^^,^^17^ Case studies exploring challenges in implementing early warning systems reported climate services training not being a core competency in public health or policy and those in health-related decision-making positions may have reduced capacity or willingness to make informed climate-related decisions.^18^

This article found that governance structures hinder cross-departmental and interagency collaboration, limiting successful implementation of climate and health adaptation. Typically ministries and departments receive independent funding to respond to their specific mandate and external consultation with public health professionals are not mandatory for decision-making, which may have negative consequences for health.^18^ The ECHO on reviewing National Adaptation Strategies and Health Adaptation Plans (HNAPS) identified that most call for cross-sectoral, interdisciplinary teams to address the climate impacts on health, however, this was not prioritized in HNAPS.^19^ Introducing and allocating resources, which promote collaborative engagement between disciplines, departments and external partners, e.g. academia may be a solution for robust, effective climate and health-adaptation policies.^14^^,^^20^^,^^21^ Finally, evidence indicates that improved access to data and the operationalization of knowledge and evidence are key levers for accelerating climate and health adaptation between agencies.^22^ A global survey of representatives from national public health associations unveiled that enriching knowledge through introducing collaborative platforms may facilitate public health progress in climate adaptation.^23^ International and national level guidance can facilitate local level action provided they include relevant, practical information and indicators to benefit health.

### What this study adds

This study presents a current view of the varied and often limited implementation of adaptation measures within the public health sector, although it is gaining attention at local and national levels. By interviewing decision-makers at different governance levels and across roles, we could observe insights into the current barriers and facilitators for effective implementation of climate and health adaptation and identify opportunities for alleviating these challenges. Currently, there is a lack of consideration of the health benefits of adaptation across departments outside of public health, as well as potential disbenefits from policies developed without consultation from health professionals. This study presents key recommendations to accelerate future implementation of climate adaptation in the public health sector (Box 1).

### Limitations of the study

We had difficulty recruiting public health participants due to our sampling approach, and most interviewees were from city-level government because of high work pressures limiting availability of interviewees from other governance levels. Therefore, the themes identified in this article may be less representative of national and international government perspectives, although insights are relevant as most implementation of interventions happens locally. Furthermore, as the study participants represented 17 EU countries and two European countries, the findings are not representative of all of Europe. This thematic analysis and coding of transcripts was carried out by a single researcher. During the development of the analytical framework, the research team consulted on themes and sub-themes identified as well as multiple researchers analysing 25% of the transcripts and comparing outputs to minimize coder bias.

## Conclusion

Across Europe, there is varied progress in the implementation of climate change adaptation for health. Providing appropriate resources, inter-departmental collaboration, training, knowledge mobilization and multi-level governance support will facilitate climate and health policy implementation. Integrating public health into all policies is key for delivering climate adaptation action which benefits health.

## Supplementary Material

ckad218_Supplementary_Data

## Data Availability

The data underlying this article cannot be shared publicly due to ensuring the privacy of individuals that participated in the study as stated in the Ethical Approval agreement with the London School of Hygiene and Tropical Medicine Ethics Committee. The data will be shared on reasonable request to the corresponding author. Box 1 Recommendations for future adaptation policy implementation for public health sector
**Collaborative approach:** All policy development should use a collaborative approach between departments, agencies and governance levels to integrate health into climate adaptation action.
**Governance restraints and policy remit:** Ensure city and national governments have established, clear remit with specific job roles to implement climate and health policies.
**Resource allocation:** Public health departments require additional funding, personnel and training to adequately address the climate and health agenda.
**Operationalizing knowledge:** Improve data access, sharing of evidence between departments and agencies and provide simple, accessible evidence platforms for ease of access.
