# Supplementary material for: Implementation of climate adaptation in the public health sector in Europe: qualitative thematic analysis
Source: Eur J Public Health. 2023 Dec 14;34(3):544–9. doi: 10.1093/eurpub/ckad218 (PMC11161147; doi:10.1093/eurpub/ckad218)
Supplement: ckad218_Supplementary_Data [file ckad218_supplementary_data.pdf]

Supplementary material

S1: Interview topic guide

Interest in climate and health	What is organisations mission and primary goal with respect to climate/health adaptation?<br><br>What is your awareness of adaptation policy for health?<br><br>What is the desired outcome of their efforts?<br><br>What motivates their work?
Influence	What is their capacity for implementation of [health adaptation]?<br><br>How strongly does your organisation influence adaptation policy and intervention?<br><br>Does your organisation have the necessary resources for implementation and ability to mobilise other groups?
Current policy gaps	Are they supportive or critical of current national policy on adaptation for health? [national or local adaptation plan/EU adaptation strategy]<br><br>How/do national policies constrain local action? [for city level only]
Network questions for mapping	Who do you regularly cooperate with in the field of climate adaptation and health?<br><br>Which organisation do you interact with? (policy, conflict, funding, organisational hierarchy, other)<br><br>Who would like to work with?
Current evidence gaps	What research evidence do you use?<br><br>What evidence would help your work?

S2: Full list of codes used in thematic analysis of interview transcripts.

Theme	Code
Barriers	Ability to mobilise groups
	Awareness raising and motivation
	Behaviour change not considered
	Climate change not considered in health strategy
	Covid-19 capacity constraints
	Collaborative working with departments, partners, public etc.
	Considering health implications
	Data access
	Delivery of agenda
	Departmental silos
	Disconnect between city and national level
	Dissemination of research to local level
	Engaging with public on policies
	Evidence needs
	Financial implications
	Governance constraints
	Grant proposals unobtainable
	Implementation capacity
	Knowledge limitations
	Lack of planning, strategy or aim, mission
	Lack of public trust
	Limitations in person capacity to address risks
	No climate change department
	Resource availability
	Scientific jargon
	Short term focus
	Spatial differences in climate strategy or target
	Surveillance or indicator needs
	Time constraints
	Too mitigation focused

Theme	Code
Facilitators	Ability to implement strategies
	Ability to mobilise other groups
	Accessible data and research
	Behaviour change importance
	Capacity and resource
	Climate/health network membership
	Climate working groups
	Collaborative working with departments or organisations
	Effective communication of findings

	Engagement with public sector
	European funding project
	Financial resource availability
	Governance structure
	Guidance and regulations
	Health importance
	Knowledge generation
	Legal mandate
	Motivation and political will
	Public acceptance or support
	Response and operational at city level
	Sharing good practice
	Strategies and plans
	Supporting local authorities

**Theme**	**Code**
Evidence Needs	Adaptation action and guidance
	Behavioural aspects or research
	Best practice from Europe
	City level risk assessments
	Climate risk analysis
	Climate risk methods
	Communication of results/policy briefs
	Data needs
	Disease trends
	Engage with local authorities
	Engage with vulnerable groups
	Engagement with private sector
	Engagement with public
	Green finance models
	Health impacts extreme events
	Health relevance in adaptation
	Indicators
	Intervention/evaluation studies
	Local level evidence
	Long term cost analysis
	Mental health impacts
	Practice and place based research
	Training needs
	Vulnerability mapping
	Other

**Theme**	**Code**
Public health role/agend a	Health in all policies
	Intersectoral working or interdepartmental collaboration
	New role created e.g. adaptation lead
	Public health responsibilities
	Wider determinants of health

S3: COREQ (Consolidated criteria for Reporting Qualitative research) completed checklist

COREQ (COnsolidated criteria for REporting Qualitative research) Checklist

A checklist of items that should be included in reports of qualitative research. You must report the page number in your manuscript where you consider each of the items listed in this checklist. If you have not included this information, either revise your manuscript accordingly before submitting or note N/A.

Topic	Item No.	Guide Questions/Description	Reported on Page No.
**Domain 1: Research team and reflexivity**			
Personal characteristics			
Interviewer/facilitator	1	Which author/s conducted the interview or focus group?	5
Credentials	2	What were the researcher's credentials? E.g. PhD, MD	5
Occupation	3	What was their occupation at the time of the study?	5
Gender	4	Was the researcher male or female?	n/a
Experience and training	5	What experience or training did the researcher have?	6
Relationship with participants			
Relationship established	6	Was a relationship established prior to study commencement?	5
Participant knowledge of the interviewer	7	What did the participants know about the researcher? e.g. personal goals, reasons for doing the research	Supplementary
Interviewer characteristics	8	What characteristics were reported about the inter viewer/facilitator? e.g. Bias, assumptions, reasons and interests in the research topic	5
**Domain 2: Study design**			
Theoretical framework			
Methodological orientation and Theory	9	What methodological orientation was stated to underpin the study? e.g. grounded theory, discourse analysis, ethnography, phenomenology, content analysis	4
Participant selection			
Sampling	10	How were participants selected? e.g. purposive, convenience, consecutive, snowball	5
Method of approach	11	How were participants approached? e.g. face-to-face, telephone, mail, email	5
Sample size	12	How many participants were in the study?	5
Non-participation	13	How many people refused to participate or dropped out? Reasons?	n/a
Setting			
Setting of data collection	14	Where was the data collected? e.g. home, clinic, workplace	5
Presence of non-participants	15	Was anyone else present besides the participants and researchers?	n/a
Description of sample	16	What are the important characteristics of the sample? e.g. demographic data, date	5, 6
Data collection			
Interview guide	17	Were questions, prompts, guides provided by the authors? Was it pilot tested?	5 & S1
Repeat interviews	18	Were repeat inter views carried out? If yes, how many?	n/a
Audio/visual recording	19	Did the research use audio or visual recording to collect the data?	5
Field notes	20	Were field notes made during and/or after the inter view or focus group?	5
Duration	21	What was the duration of the inter views or focus group?	5
Data saturation	22	Was data saturation discussed?	5
Transcripts returned	23	Were transcripts returned to participants for comment and/or	n/a

Topic	Item No.	Guide Questions/Description	Reported on Page No.
		correction?	
**Domain 3: analysis and findings**			
Data analysis			
Number of data coders	24	How many data coders coded the data?	5
Description of the coding tree	25	Did authors provide a description of the coding tree?	S2
Derivation of themes	26	Were themes identified in advance or derived from the data?	5,6
Software	27	What software, if applicable, was used to manage the data?	5,6
Participant checking	28	Did participants provide feedback on the findings?	n/a
Reporting			
Quotations presented	29	Were participant quotations presented to illustrate the themes/findings? Was each quotation identified? e.g. participant number	6 - 11
Data and findings consistent	30	Was there consistency between the data presented and the findings?	6 - 11
Clarity of major themes	31	Were major themes clearly presented in the findings?	6 - 11
Clarity of minor themes	32	Is there a description of diverse cases or discussion of minor themes?	6 - 11

Developed from: Tong A, Sainsbury P, Craig J. Consolidated criteria for reporting qualitative research (COREQ): a 32-item checklist for interviews and focus groups. *International Journal for Quality in Health Care*. 2007. Volume 19, Number 6: pp. 349 – 357

Once you have completed this checklist, please save a copy and upload it as part of your submission. DO NOT include this checklist as part of the main manuscript document. It must be uploaded as a separate file.

S4: Participant information sheet and consent form

Stakeholder analysis of policy makers within European region for climate and health adaptation

Connecting HEALTH & CLIMATE CHANGE

**Statement**	**Please initial each box**
I confirm that I have read and understood the information sheet for the ENBEL study on climate and health adaptation. I have had the opportunity to consider the information, ask questions and have these answered satisfactorily.	
I understand that my consent is voluntary and that I am free to withdraw this consent at any time without giving any reason.	
I understand that relevant sections of my data collected during the study may be looked at by authorised individuals from the London School of Hygiene and Tropical Medicine, Dipartimento di Epidemiologia del S.S.R Lazio, and the University of Graz, where it is relevant to my taking part in this research. I give permission for these individuals to have access to these data.	
I understand that data about me may be shared via a public data repository or by sharing directly with other researchers, and that I will not be identifiable from this information	
I agree to me taking part in the above-named study.	

Printed name of participant	Signature of participant	Date

Printed name of person obtaining consent	Signature of person obtaining consent	Date

Stakeholder analysis of policy makers within European region

for climate and health adaptation

Connecting HEALTH & CLIMATE CHANGE

We would like to invite you to take part in a research study on implementation of adaptation strategies to protect human health.

What is the purpose of the study?

The ENBEL project (Enhancing Belmont) aims to support policy making in the EU and Europe on climate and health. ENBEL brings together leaders in climate change and health research through a network of international research projects funded under the Belmont Forum's Collaborative Research Action (CRA), Societal Challenge 1 and 5 of EU's Horizon 2020. ENBEL will develop evidence syntheses and co-produce with stakeholders targeted knowledge products. We aim to map stakeholders in Europe that work on adaptation to reduce the health impacts of climate change in order to understand interlinkages and identify areas for action. ENBEL is funded by the European Commission.

Why have I been asked to take part?You have been identified as a key stakeholder for climate adaptation for health policy.

Do I have to take part?

It is up to you to decide to take part or not. Nothing will happen if you do not take part in this study. You can withdraw from the study at any time.

What will I have to do?

Your participation consists of a single interview that will be recorded and transcribed. All results will be fully anonymised and no quotes will be attributed to you. Your participation is voluntary and you can withdraw from the project at any time. The interview should take about 40 minutes.

What are the possible risks and disadvantages?

We are not aware of any risks or disadvantages of participating in this research.

What are the possible benefits?

We cannot promise the study will help you but the information we get from the study will help our knowledge and understanding of the implementation of adaptation measures on climate and health adaptation and will also help us to identify research needs in this area.

If you have concerns

If you have a concern about any aspect of this study, you can speak to the researchers who will do their best to answer your questions <contact number>. If you remain unhappy and wish to complain formally, you can do this by contacting our research grants officer at LSHTM: Patricia Henley at rgio@lshtm.ac.uk or +44 (0) 20 7927 2626. The London School of Hygiene and Tropical Medicine holds insurance policies which apply to this study. If you experience harm or injury as a result of taking part in this study, you may be eligible to claim compensation.

What will happen to information collected about me?

We will need to use information from your interview for this research project. All information collected about you will be kept private. Only the study staff and authorities who check that the study is being carried out properly will be allowed to look at the information. Data may be sent to other study staff in ENBEL but this will be anonymised.

Your personal details, meaning your name and other identifiable information, will be kept in a different safe place to the other study information and will be destroyed within 10 years of the end of the study. At the end of the project, the study data will be archived at LSHTM.

Where can you find out more about how your information is used?

You can find out more about how we use your information at https://www.lshtm.ac.uk/files/research-participant-privacy-notice.pdf or by asking one of the research team or by sending an email to DPO@lshtm.ac.uk

What will happen to the results of this study?

The results of this research will be delivered to the European Commission and research funding agencies to inform the future research agenda on climate and health. We will also publish the results in a research journal.

Who is organising and funding this study?

London School of Hygiene & Tropical Medicine is the sponsor for the research and they have full responsibility for the project including the collection, storage and analysis of your data, and will act as the Data Controller for the study. This means that we are responsible for looking after your information and using it properly. The research protocols have been reviewed and given favourable opinion by The London School of Hygiene and Tropical Medicine Research Ethics Committee (ref: 25707).

Thank you

Thank you for taking time to read this information sheet. If you think you will take part in the study, please read and sign the consent form. If you would like any further information, please contact Dr Sari Kovats (sari.kovats@lshtm.ac.uk) who can answer any questions you may have about the study.
